# Training text chunkers on a silver standard corpus: can silver replace gold?

**DOI:** 10.1186/1471-2105-13-17

**Published:** 2012-01-30

**Authors:** Ning Kang, Erik M van Mulligen, Jan A Kors

**Affiliations:** 1 Department of Medical Informatics, Erasmus University Medical Center, P.O. Box 2040, 3000 CA Rotterdam, The Netherlands

## Abstract

**Background:**

To train chunkers in recognizing noun phrases and verb phrases in biomedical text, an annotated corpus is required. The creation of gold standard corpora (GSCs), however, is expensive and time-consuming. GSCs therefore tend to be small and to focus on specific subdomains, which limits their usefulness. We investigated the use of a silver standard corpus (SSC) that is automatically generated by combining the outputs of multiple chunking systems. We explored two use scenarios: one in which chunkers are trained on an SSC in a new domain for which a GSC is not available, and one in which chunkers are trained on an available, although small GSC but supplemented with an SSC.

**Results:**

We have tested the two scenarios using three chunkers, Lingpipe, OpenNLP, and Yamcha, and two different corpora, GENIA and PennBioIE. For the first scenario, we showed that the systems trained for noun-phrase recognition on the SSC in one domain performed 2.7-3.1 percentage points better in terms of F-score than the systems trained on the GSC in another domain, and only 0.2-0.8 percentage points less than when they were trained on a GSC in the same domain as the SSC. When the outputs of the chunkers were combined, the combined system showed little improvement when using the SSC. For the second scenario, the systems trained on a GSC supplemented with an SSC performed considerably better than systems that were trained on the GSC alone, especially when the GSC was small. For example, training the chunkers on a GSC consisting of only 10 abstracts but supplemented with an SSC yielded similar performance as training them on a GSC of 100-250 abstracts. The combined system even performed better than any of the individual chunkers trained on a GSC of 500 abstracts.

**Conclusions:**

We conclude that an SSC can be a viable alternative for or a supplement to a GSC when training chunkers in a biomedical domain. A combined system only shows improvement if the SSC is used to supplement a GSC. Whether the approach is applicable to other systems in a natural-language processing pipeline has to be further investigated.

## Background

Chunking is a natural language processing technique that splits text into groups of words that constitute a grammatical unit, e.g., a noun phrase or a verb phrase. It is an important processing step in systems that try to automatically extract information from text. Most chunkers are based on machine learning methods and require a text corpus annotated with chunks for training the system. The creation of a gold standard corpus (GSC) is tedious and expensive: annotation guidelines have to be established, domain experts must be trained, the annotation process is time-consuming, and annotation disagreements have to be resolved. As a consequence, GSCs in the biomedical domain are generally small and focus on specific subdomains, which limit their usefulness.

In this study we investigate an alternative, automatic approach to create an annotated corpus. We have shown before that a system combining the outputs of various chunkers performs better than each of the individual chunkers. Here we postulate that the annotations of such a combined system on a given corpus can be taken as a reference standard, establishing a "silver standard corpus" (SSC).

To test the practical value of this approach, we explore two use scenarios of such an SSC. In the first scenario, a chunker has to be trained for a biomedical subdomain for which a GSC is not available. Rather than creating a new GSC, we generate an SSC for the new domain and train the chunker on the SSC. In the second scenario, a GSC from the domain of interest is available but its size is small and a chunker trained on it gives suboptimal performance. Rather than expanding the GSC, we supplement the GSC with an SSC from the same domain and train the chunker on the combined GSC and SSC to improve chunker performance.

### Related work

During the past decade, much research has been devoted to systems that combine different classifiers, also called multiple classifier systems or ensemble-based systems [1]. The general idea is that the combined wisdom of multiple classifiers reduces the risk of errors, and indeed it has been shown many times that a combined system performs better than the best individual classifier. Multiple classifier systems have been applied in many domains, including biomedical text mining and information extraction. For instance, Smith et al. [2] combined the results of 19 systems for gene mention recognition, and found that the combined system outperformed the best individual system by 3.5 percentage points in terms of F-score. Kim et al. [3] combined eight systems for event extraction and showed that the performance of the combined system increased by 4 percentage points as compared to the best individual system. We previously combined six publicly available text chunkers using a simple voting approach [4]. The F-score of the combined system improved by 3.1 percentage points for noun-phrase recognition and 0.6 percentage point for verb-phrase recognition as compared to the best single chunker.

The notion that a combination of systems can be used to create a "silver standard" corpus has been explored in the CALBC (Collaborative Annotation of a Large Biomedical Corpus) project [5]. Through CALBC, the natural-language processing community has been invited to annotate a very large biomedical corpus with a variety of named-entity recognition systems. The combined annotations of multiple systems may provide a valuable resource for system development and evaluation, and the automatically generated creation of an SSC would allow corpora of unprecedented size. In a very recent study, Chowdhury and Lavelli compared a gene recognition system trained on an initial version of the CALBC SSC against the system trained on the BioCreative GSC [6]. The system trained on the SSC performed considerably worse than when trained on the GSC, but the authors propose several ways to automatically improve the quality of the SSC and are of the opinion that, in the absence of a GSC, a system trained on the SSC could be useful in the semi-automatic construction of a GSC.

## Methods

### Chunking systems

To generate a silver standard, we used five well-known and publicly available chunkers: GATE chunker 5.0 [7], Lingpipe 3.8 [8], MetaMap 2008v2 [9], OpenNLP 2.1 [10], and Yamcha 0.33 [11]. Three of these chunkers are trainable (Lingpipe, OpenNLP, Yamcha), the other two do not have a training option. Sentence splitting, tokenization, and part-of-speech tagging were included in our chunking pipeline, either as integral part of the chunkers (Yamcha, Lingpipe) or as separate components (OpenNLP). We used the gold-standard sentence, token, and part-of-speech annotations for training, but did not use this information in creating the SSC or evaluating the trained models: the input of the annotation pipeline consisted of plain abstracts, the output were chunking annotations. All chunkers annotate noun phrases and verb phrases, except for GATE which only generates noun phrases. More information on characteristics and performance of these chunkers can be found in our previous comparative study of chunkers [4], which also included Genia Tagger. Since Genia Tagger comes with a fixed pre-trained model based on the corpora that we use in this study, it could bias the results of our experiments and was not included. All chunkers were used with default parameter settings.

### Corpora

There are only a few publicly available corpora in the biomedical domain that incorporate chunk annotations. We used the GENIA Treebank corpus [12] and the PennBioIE corpus [13].

The GENIA corpus [12] has been developed at the University of Tokyo. The 1.0 version of the corpus was released in 2009 and consists of 1,999 Medline abstracts selected from a query using the MeSH terms "human", "blood cells", and "transcription factors". The corpus has been annotated with various levels of linguistic and semantic information, such as sentence splitting, tokenization, part-of-speech tagging, chunking annotation, and term-event information. For chunker training, we selected a subset of 500 abstracts that constituted a previous version of the GENIA corpus [12].

The PennBioIE Treebank corpus [13] has been developed at the University of Pennsylvania. The 0.9 version of the corpus was released in 2004 and includes the CYP and Oncology corpora of the Linguistic Data Consortium. The CYP corpus consists of 324 Medline abstracts on the inhibition of cytochrome P450 enzymes. The Oncology corpus consists of 318 Medline abstracts on cancer and molecular genetics. The corpus has been tokenized and annotated with paragraph, sentence, part-of-speech tagging, chunking annotation, and biomedical named-entity types.

### Creation of the silver standard

We used a simple voting scheme to generate silver standard annotations from the annotations produced by the different chunkers. For each phrase identified by a chunker, the number of chunkers that gave exactly matching annotations was counted. If the count was larger than or equal to a preset voting threshold, the phrase was considered a silver standard annotation, otherwise it was not. In all our experiments, we used a voting threshold of three out of five chunkers for noun phrases, and a threshold of two out of four for verb phrases (GATE only generates noun phrases). These thresholds gave uniformly the best results in terms of F-score when the silver standard annotations of the training data were evaluated against the gold standard. The Unstructured Information Management Architecture (UIMA) framework [14] was used to integrate all chunking systems and combine their result.

### Silver standard as alternative for gold standard

To test whether an SSC could serve as a substitute for a GSC, we compared the performance of chunkers trained on silver standard annotations of the abstracts in the PennBioIE corpus with the performance of the chunkers trained on the gold standard annotations of the same corpus. To create the SSC, the trainable chunkers (Lingpipe, OpenNLP, Yamcha) were trained on the gold standard annotations of 500 abstracts of the GENIA corpus. The chunkers then annotated the PennBioIE corpus and the annotations of all chunkers were combined to yield the silver standard. Subsequently, Lingpipe, OpenNLP, and Yamcha were trained on the PennBioIE SSC and on the PennBioIE GSC, using 10-fold cross-validation. In the cross-validation procedure for the SSC, the annotations of the abstracts in each test fold were taken from the GSC. Thus, the performance of chunkers trained on either SSC or GSC was always tested on the GSC.

### Silver standard as supplement of gold standard

To test whether an SSC would have additional value as a supplement for a given GSC, we compared the performance of chunkers trained on a subset of the GENIA GSC with the performance of the chunkers trained on the same subset supplemented with an SSC. Specifically, subsets of 10, 25, 50, 100, and 250 abstracts were selected from the initial GENIA training set of 500 abstracts, each subset being contained in the next larger one. Lingpipe, OpenNLP, and Yamcha were trained on the gold standard annotations of each subset and the total set, and tested on the 1,499 GENIA abstracts that were not used for training. For each subset, the chunkers trained on that subset were subsequently used to create an SSC of the abstracts in the set of 500 abstracts that were not part of the subset, i.e., for the GSC subset of 10 abstracts, the SSC consisted of the remaining 490 abstracts; for the subset of 25 abstracts, the SSC consisted of 475 abstracts; etc. The GSC and corresponding SSC (together always totaling 500 abstracts) were then used to train the chunkers. Their performance was tested again on the 1,499 GENIA abstracts not used for training. The above experiment was repeated 10 times, each time starting with a different randomly selected subset of 10 abstracts. The reported results are the averaged F-scores of the 10 experiments.

### Performance evaluation

The chunker and silver standard annotations were compared with the gold standard annotations by exact matching, similar to the procedure followed in CoNLL-2000 [15]. An annotation was counted as true positive if it was identical to the gold standard annotation, i.e., both annotations had the same start and end location in the corpus. A phrase annotated by the gold standard was counted as false negative if the system did not render it exactly; a phrase annotated by a system was counted as false positive if it did not exactly match the gold standard. Performance of the chunkers and silver standard was evaluated in terms of precision, recall, and F-score.

To reduce the effect of insignificant differences between chunks, words from the stopwords list in PubMed [16] and punctuation remarks were removed before matching if they appeared at the start or the end of a phrase. For instance, "[the protein's binding site on the DNA molecule]NP is..." is considered the same annotation as "the [protein's binding site on the DNA molecule]NP is...", and "the medicine [often causes]VP..." is considered the same as "the medicine often [causes]VP...".

## Results

### Silver standard as alternative for gold standard

Table 1 shows the performance of the three trainable chunkers and the combined system on the PennBioIE GSC when trained on three different corpora: GENIA GSC, PennBioIE SSC, or PennBioIE GSC. GATE and MetaMap could not be trained and when tested on the PennBioIE GSC had F-scores of 78.2% (MetaMap) and 72.8% (GATE) for noun phrases, and 77.7% (MetaMap) for verb phrases. Clearly, the trainable chunkers perform better if they are trained on the PennBioIE SSC than on the GENIA GSC. The increase in F-scores varies between 1.7 and 3.1 percentage points for noun phrases and between 1.0 and 3.3 percentage points for verb phrases. Although performance further increases when training on PennBioIE GSC instead of PennBioIE SSC, differences are not large: 0.2 to 0.8 percentage point for noun phrases, 0.3 to 1.7 percentage point for verb phrases. OpenNLP consistently shows the best performance both for noun and verb phrases. The combined system performs better than any of the individual chunkers, including GATE and MetaMap which proved to have F-scores lower than each of the three trainable chunkers, in agreement with our previous findings [4]. The largest improvement of the combined system is seen when the individual chunkers are trained on the GENIA GSC. Remarkably, the performance difference between the combined systems based on GENIA GSC and PennBioIE SSC is only small (0.2 percentage point). To test the consistency of this result, we redid the experiment with interchanged corpora, i.e., GENIA GSC was used for training the chunkers and generating the SSC, and PennBioIE GSC was used for testing. The F-score of the combined system by using GENIA SSC for training was 0.5 (noun phrases) and 0.4 (verb phrases) percentage point better than the F-score of the combined system by using PennBioIE GSC for training, which is comparable with the results of the initial experiment.

**Table 1:** Performance (F-score) of chunkers and their combination when trained for noun-phrase and verb-phrase recognition on different training sets.

	Training set for noun phrases			Training set for verb phrases		
	
System	GENIA GSC	PennBioIE SSC	PennBioIE GSC	GENIA GSC	PennBioIE SSC	PennBioIE GSC
Lingpipe	75.8%	78.5%	78.7%	90.6%	91.6%	91.9%
OpenNLP	80.8%	83.9%	84.7%	90.7%	93.2%	94.8%
Yamcha	80.1%	83.2%	84.0%	89.5%	92.8%	94.2%
Combined	84.3%	84.5%	87.2%	93.7%	93.9%	95.5%

All systems are tested on the PennBioIE corpus.

### Silver standard as supplement of gold standard

Table 2 shows the performances of chunkers and the combined system when trained on GSCs of varying sizes and on the GSCs supplemented with an SSC. For all sizes of the GSC, the systems trained on a combination of GSC and SSC always perform better than the systems trained on the GSC alone. Clearly, the improvement is largest for small sizes of the GSC, leveling off with increasing size. The performance obtained with a small set of GSC abstracts combined with an SSC is comparable to a larger GSC set without SSC. For instance, each system trained on a GSC of only 10 abstracts supplemented with the SSC performs better than the system trained on a GSC of 100 abstracts alone; For larger GSC sizes, the performance of OpenNLP or Yamcha trained on 100 or 250 GSC abstracts plus the SSC is within 1 percentage point of the performance of the system trained on the next larger size of the GSC alone (250 and 500 abstracts, respectively).

**Table 2:** Performance (F-score) of chunkers and their combination trained on subsets of different size of the GENIA GSC and on the GSC subset supplemented with an SSC, for noun-phrase and verb-phrase recognition.

	Lingpipe		OpenNLP		Yamcha		Combined	
				
GSC size	GSC	GSC+SSC	GSC	GSC+SSC	GSC	GSC+SSC	GSC	GSC+SSC
Noun phrases								
10	65.8%	80.8%	83.0%	87.9%	82.7%	85.6%	86.8%	90.7%
25	72.2%	81.1%	85.7%	88.3%	84.3%	86.0%	87.9%	90.9%
50	76.8%	81.3%	87.5%	88.6%	85.4%	86.2%	88.9%	91.2%
100	78.2%	81.9%	87.9%	88.9%	85.6%	86.6%	89.3%	91.5%
250	82.4%	82.8%	88.3%	89.3%	86.7%	87.2%	90.6%	92.0%
500	84.5%	n.a	89.7%	n.a	88.1%	n.a	92.8%	n.a
Verb phrases								
10	64.1%	86.9%	84.3%	93.6%	86.2%	92.5%	91.3%	94.6%
25	73.8%	87.3%	88.8%	94.0%	89.7%	92.9%	93.0%	94.9%
50	79.2%	87.6%	92.1%	94.4%	91.7%	93.1%	94.4%	95.5%
100	83.6%	87.9%	93.6%	94.7%	92.3%	93.4%	95.4%	95.8%
250	88.3%	88.7%	95.0%	95.3%	93.8%	93.9%	95.8%	96.0%
500	90.3%	n.a	95.7%	n.a	94.1%	n.a	96.3%	n.a

## Discussion

We have investigated the use of an SSC as a substitute or a supplement of a GSC for training chunkers in the biomedical domain. The SSC as a substitute for a GSC corresponds with a use scenario in which a chunker created for one subdomain has to be adapted to another, where a GSC for the new domain is not available. We have shown that a system trained on an SSC for the new domain performs considerably better than if that system is trained on the GSC of another subdomain, and only slightly worse (< 1 percentage point) than if the system was trained on a GSC for the new domain. In the second use scenario, we supplemented a (small) GSC with an SSC for the same domain as the GSC. The addition of the SSC always improved the chunker performance, particularly if the size of the initial GSC was small.

Our results on the practical value of an SSC are different from those that were recently reported by Chowdhury and Lavelli [6]. They found a considerable drop in performance of a gene recognition system trained on the CALBC SSC as compared to the system trained on the BioCreative GSC, and also noticed that the system trained on a combination of SSC and GSC performed worse than on the GSC only. There may be several reasons for these differences. One is that the SSC that we used for training the chunkers was evaluated against the GSC of the same subdomain, whereas in the other study the domains from which the CALBC SSC and the BioCreative GSC are taken, are more divergent. Another possible reason is that the quality of the CALBC SSC is simply not good enough, which may be related to the difficulty of the CALBC task. Named entity recognition is generally considered more difficult than chunking, having to deal with increased complexities in boundary recognition, disambiguation, and spelling variation of entities. Clearly, the better a silver standard will approach a gold standard for the domain of interest, the better the performance of systems trained on an SSC. It should be noted that the performance of the silver standard compared with the gold standard in our study is far from perfect: the PennBioIE SSC has an F-score of 84.5% for noun phrases and 93.9% for verb phrases. Performance figures of the CALBC SSC against GSCs for named-entity recognition are not yet available, but we presume that they will be much lower. However, despite the differences between an SSC and GSC, chunking systems trained on these corpora showed remarkably similar performances. It is still an open question how an SSC of lower (or higher) quality affects the performance of a system trained on the SSC.

We used a simple voting approach to create an SSC. More sophisticated voting methods exist, such as weighted voting [17] or Borda count [18], but these methods require information about the confidence or rank of the chunks, information that is not available for the chunkers in this study. We also tested a combined system based on the output of the three trainable chunkers instead of all five chunkers. When trained on GENIA GSC and tested on PennBioIE GSC, the F-score of the combined system dropped to 82.1% for noun phrases and 91.9% for verb phrases. Since this performance is considerably lower than that of the combined system based on all chunkers, we did not further pursue the use of an SSC based on the three trainable chunkers only.

We used exact matching in performance assessment of the chunkers and creation of the SSC. By removing stopwords before matching we tried to remove "uninformative" words that should not play a role in determining whether phrases are the same, similar to other studies (e.g., [19,20]). Our main consideration to remove stopwords was that chunking is usually an intermediate step in the information extraction pipeline, and whether an unimportant word (e.g., "the" at the start of a noun phrase) is detected or not, is unlikely to affect subsequent processing steps (e.g., named entity recognition). Stopword removal can be seen as a relaxation of the strict matching requirement. When systems trained on GENIA GSC were tested on PennBioIE GSC but without removing stopwords, performances dropped by 3.7-5.5 percentage points for noun phrases and 3.6-6.3 percentage points for verb phrases. This shows that chunkers may considerably differ with the gold standard with respect to the annotation of stopwords. We did not want to further relax the matching criterion, e.g., by allowing partially matching boundaries, first because this would produce matches between phrases that differ in other than uninformative words (and thus should be considered different), and second because it is not obvious how partially matching phrases should be combined in a single phrase for inclusion in the SSC.

Since the creation of an SSC is automatic, its size can be very large. For different text-processing applications, increasing amounts of data for training classifiers have been shown to improve classifier performance [21-23]. Use of an SSC may be beneficial in mitigating the "paucity-of-data" problem [21].

The combination of systems always performed better than any of the individual systems, but performance increase of the combined system was larger when the individual systems were trained on GENIA or PennBioIE GSCs than when they were trained on the PennBioIE SSC (cf. Table 1). A possible explanation for this phenomenon is that the SSC incorporates results from the chunkers that are subsequently trained on it. As a consequence, the diversity of the chunkers trained on the SSC may be less than those trained on the GSCs. Indeed, when we pairwise determined the F-score between two chunkers trained on GENIA GSC and PennBioIE GSC, the average score was 78.2% and 80.2%, respectively, in comparison to 87.4% for PennBioIE SSC (without stopword removal these figures were 72.6%, 73.9%, and 82.5%, respectively). This indicates better agreement between the chunkers (less diversity) for the SSC. Since annotation diversity is generally considered a key factor for the improvement seen by ensemble systems (4), it may be expected that the combined chunker system shows a smaller increase of performance when based on the SSC than on the GSCs.

We showed that chunkers can obtain almost similar performances whether trained on an SSC or a GSC, but this does not mean that we can dispose of GSCs altogether. Obviously, to create the SSC we need trained chunkers, and thus a GSC for their initially training. We explored the use of a GSC from another, but related, domain than the domain of interest. Alternatively, we supplemented a GSC with an SSC in the same domain of interest. Using this approach, good results can be achieved with remarkably small-sized GSCs. Our experiments indicated that a GSC consisting of only 10 or 25 abstracts but expanded with an SSC yields similar performances as a GSC of 100 or 250 abstracts. Practically, these results suggest that the time and effort spent in creating a GSC of sufficient size may be much reduced.

We have tested two use scenarios of an SSC in the field of text chunking, but the proposed approach is general and could be used in any field in which GSCs are needed to train classifiers. Further investigations will have to reveal how the quality of an SSC affects classifier performance and whether the use of SSCs in other application areas is equally advantageous as their use in text chunking.

## Conclusions

We have shown that an automatically created SSC can be a viable alternative for or a supplement to a GSC when training chunkers in a biomedical domain. A combined system only shows improvement if the SSC is used to supplement a GSC. Our results suggest that the time and effort spent in creating a GSC of sufficient size may be much reduced. Whether the approach is applicable to other systems in a natural-language processing pipeline has to be further investigated.

## Authors' contributions

NK co-developed the methodology, built the software infrastructure, carried out the experiments, and drafted the manuscript. EMM and JAK conceived the study, co-developed the methodology, and helped to write the paper. All authors read and approved the manuscript.
